# Correlates of food insecurity among university students in a socioeconomically disadvantaged area of the Paris suburbs: A cross-sectional study

**DOI:** 10.1371/journal.pone.0334523

**Published:** 2025-10-29

**Authors:** Henri Dehove, Malo Mofakhami, Julia Baudry, Emmanuelle Kesse-Guyot, Chantal Julia, Mathilde Touvier, Sandrine Péneau, Alice Bellicha

**Affiliations:** 1 Université Sorbonne Paris Nord and Université Paris Cité, INSERM, INRAE, CNAM, Center of Research in Epidemiology and StatisticS (CRESS), Nutritional Epidemiology Research Team (EREN), Bobigny, France; 2 Institute for Interdisciplinary Research on Social Issues (IRIS), Center for employment and labor studies (CEET), Université Sorbonne Paris Nord, Villetaneuse, France; 3 Public Health Department, Avicenne Hospital, Assistance Publique des Hôpitaux de Paris (AP-HP), Bobigny, France; 4 Institut Universitaire de France (IUF), Paris, France; University of Petra (UOP), JORDAN

## Abstract

**Background:**

Despite increasing political and scientific interest in food insecurity (FI) among higher education students, data for Europe remain scant. We assessed the prevalence of FI at a French university located in a disadvantaged area in the outskirts of Paris, and its associations with perceived academic dropout and socioeconomic, demographic, and lifestyle factors.

**Methods:**

We conducted a cross-sectional online survey among 5068 students (22% of overall population, 66% women). FI status was defined by a three-level class variable (quantitative FI: “not having access to enough food”, qualitative FI: “not having access to the desired food”, food security: “having access to enough desired food”). Multivariate multinomial logistic regression models found associations between (i) FI and academic dropout and (ii) socioeconomic, demographic, academic-related data, cooking and eating conditions, and FI.

**Results:**

Students reporting quantitative FI (11%) or qualitative FI (35%) more often experienced academic dropout (*p* < 0.0001). Men more often reported quantitative FI (*p* < 0.0001). Living in a collective residence, lacking sufficient household cooking facilities, experiencing financial difficulties, using food assistance, being an undergraduate, having obtained a high-school diploma abroad, not receiving food from family, regularly eating alone, and infrequent cooking were positively associated with both quantitative and qualitative FI (*p* < 0.0001).

**Conclusion:**

We found elevated rates of FI among university students and an association between FI and academic dropout. Structural and behavioural factors were found to be associated with FI. These findings provide insight into the characteristics of those students most likely to experience FI and suggest testable preventive actions.

## Introduction

The Food and Agriculture Organization defines food insecurity (FI) as the lack of “regular access to enough safe and nutritious food for normal growth and development and an active and healthy life” [1]. FI, with a worldwide prevalence of 29.6% (i.e., affecting 2.4 billion people) [2], is associated with adverse physical and mental health outcomes, including weight abnormalities [3], cardiometabolic diseases [4], and mental health disorders [5]. FI is therefore a global public health issue, food security achievement being the second of the United Nations 17 Sustainable Development Goals [6]. FI is more prevalent in low-income countries [2], but its levels can also be of concern in high-income countries, especially in socioeconomically disadvantaged households [7] and in specific populations, such as higher education students [8].

Higher education students face various life changes that challenge their habits and can cause hardships, such as leaving their parental home, and meeting increased living expenses, higher academic pressure, and irregular work schedules [9]. The COVID-19 pandemic amplified these difficulties, worsening students’ financial situation [10], and increasing their risk of FI [11]. Worldwide, the higher education student population is experiencing high and increasing rates of FI [8,12,13]. For instance, in the US, recent literature reviews report an FI prevalence among students of approximately 40% [8,13], higher than the national average, evaluated at 10% [14]. Like in the general population [2], FI has been associated among students with unhealthy dietary [13] and other lifestyle habits (e.g., lower physical activity) [15], poorer physical health outcomes [13], poorer mental health outcomes such as high perceived stress [15], and more depressive symptoms [15]. Specifically in the student population, FI has been associated with negative academic outcomes, increasing the burden of FI in this population [12]. Given the associations of FI with students’ health and academic performance, FI can be viewed as a vicious circle aggravating existing social inequalities [16].

In the US, FI prevalence ranges from 10% to 58%, varying by university [12]. In France, where this study was conducted, observed FI prevalence rates ranged from 18% (Rouen, *n* = 3508) [17] to 43% (Grenoble, *n* = 4410) [18] in mainland France, and up to 75% in one French overseas region (French Guiana, *n* = 286) [19]. These variations suggest that FI prevalence may differ according to universities’ geographic and socioeconomic contexts. Besides these upstream influences, FI may be influenced by individual and interpersonal factors. Literature reviews have shown that being a woman [20], being a first-generation college student [20], coming from a low-income background [12], being financially independent from parents [13], or being an international student [21] are associated with greater FI. These associations, however, have been primarily investigated in the US, as emphasized in recent literature reviews [12,13,20], and rarely in Europe [22–24], where educative systems and student-oriented social aids differ. Besides these well-documented socioeconomic factors, other potentially important variables, such as household composition, cooking facilities, cooking habits, and use of food assistance, have not been studied in relation to FI among students. Identifying student groups most likely to experience FI is a critical first step in designing and conducting targeted preventive actions.

To address this gap in the literature, the present cross-sectional study set out (i) to evaluate the prevalence of FI in a French university located in a socioeconomically disadvantaged area, (ii) to assess the associations of FI with perceived academic dropout, and (iii) to determine the socioeconomic, demographic, and academic factors, and the cooking and eating conditions associated with FI in this population.

## Methods

### Study setting and population

This cross-sectional study was conducted at the University Sorbonne Paris Nord (USPN), located in Seine-Saint-Denis (an administrative French district area), in Paris region (France). The Seine-Saint-Denis differs from the broader Parisian region by a higher rate of unemployment [25] and a higher proportion of households living in poverty [26]. A web-based survey was administered between 1 November 2022 and 31 January 2023. All students (*n* = 22,669) enrolled at USPN at the time of the study were invited to respond by email. The survey was conducted by the University administration in accordance with the rules of the General Data Protection Regulation (GDPR) and the declaration of Helsinki. Data were collected anonymously. Participants provided written informed consent at the beginning of the study. Because the data transferred to the research team were anonymized, this study did not require approval from an ethics committee, as confirmed by the University Sorbonne Paris Nord Research Ethics Committee. The protocol was registered with the Data Protection Officer.

### Data collection

#### Food insecurity.

The questionnaire is presented in Table S1. FI was evaluated with a question previously used in the 2008 Nutrition and Health Barometer [27], translated from the Food Sufficiency Screener, which is a validated question of the U.S. Household Food Security Survey Module (HFSSM) [28]. Participants were asked to choose the item best describing their situation, out of: “I have enough of the kinds of food I want to eat” (food security), “I have enough, but not always the kinds of food I want” (qualitative FI), and “I have often or sometimes not enough to eat” (quantitative FI). FI was classified into three categories: food security, qualitative FI, and quantitative FI.

#### Socioeconomic and demographic data.

Sociodemographic data included students’ gender, whether they were living with their parents, their accommodation type (“At their parents” house”, “Living alone”, “Flat sharing”, “Collective residence”) [29], and their household cooking facilities (“No food heating equipment”, “Food heating equipment only”, “Sufficient cooking equipment”) [30]. Socioeconomic data included how often students had used food assistance since the beginning of the academic year, and whether they had financial difficulties or student jobs. Financial difficulties were assessed on a five-point Likert scale (from 1: “No financial difficulty” to 5: “Severe difficulties” [29]. “Student job” assessed whether students had a job, and if so the time spent at this job per week (“Less than 10 hours per week”, “Between 10 and 20 hours per week”, “More than 20 hours per week”) [29].

#### Academic-related data.

Students were asked whether it was their first enrolment at this university, whether they were undergoing initial training (neither apprentices nor professionals resuming studies), whether they had obtained their high-school diploma in France or abroad, their academic discipline, their study level, and whether they were experiencing academic dropout [29].

#### Cooking and eating conditions.

Students were asked whether they usually ate meals alone or with others, how often they received food from their family, and how often they had cooked in the last 15 days [27,29,30].

### Statistical analysis

Analyses included only participants without missing data. Characteristics of the included and excluded participants were compared using chi-squared tests. To improve sample representativeness compared to the overall USPN student population and thus improve the estimation of FI prevalence among this specific population, weighting was calculated using gender, study level, academic discipline, country of high school diploma attainment, and training status. The calibration weights were calculated in SAS® (version 9.4), using the “*CalMar*” macro from the French National Institute for Statistics and Economic Studies (INSEE) [31].

Descriptive comparisons between categories of FI status were performed using chi-squared tests.

Associations between FI (independent variable) and perceived academic dropout (dependent variable) were assessed using a multivariable logistic regression model, adjusted for socioeconomic and demographic data (gender, living with parents, household cooking facilities, using food assistance, financial difficulties, student job), academic-related data (first enrolment at university, undergoing initial training, high-school diploma abroad, academic discipline, study level), and cooking and eating conditions (company during meals, receiving food from family, cooking frequency).

Associations between socioeconomic, demographic and academic-related data, cooking and eating conditions, and FI status (considered as the dependent variable with three modalities, food security as reference) were assessed using a multivariable multinomial logistic regression model. For each independent variable, models were mutually adjusted for other studied variables, except for the variables “living with parents” and “accommodation type”, which overlapped and were not included simultaneously.

Given known gender differences in FI prevalence [32], interactions with gender were tested. For two variables (undergoing initial training and student job), a qualitative interaction was found with gender (i.e., when both the magnitude and direction of each variable’s effect differed by gender). Gender-stratified analyses were therefore conducted for these two variables.

All tests were two-sided, and *p* < 0.05 was considered statistically significant. Data management and statistical analyses were performed in 2024 using SAS® software (version 9.4; SAS Institute, Inc., Cary, NC, USA).

## Results

Of the 7002 students who completed the questionnaire, 1934 were excluded from the analyses because of at least one missing value, leaving a total of 5068 participants for analysis (66% women, 22% of the overall USPN student population). Excluded participants differed in FI status, with included participants more often reporting quantitative and qualitative FI (12% vs. 11% and 37% vs. 30%, respectively, *p* < 0.001) (Table S2). They also differed in socioeconomic and demographic data, academic-related data, and in cooking and eating conditions. For example, included participants were more likely to be women, to have obtained their high-school diploma abroad, to be post-graduate and PhD students, and to report financial difficulties (all *p* < 0.05).

Table 1 gives the characteristics of the weighted sample according to FI status. Table S3 gives the characteristics of participants before weighting. In the overall weighted sample, students were mostly undergraduates undergoing initial training who had obtained their high-school diploma in France. Overall, 35% and 11% of participants reported qualitative and quantitative FI, respectively.

**Table 1:** Characteristics of the sample of university students (n = 5068), by class of food insecurity.

	Weighted data ^a^^,^^b^			
	All N = 5068	Food security N = 2733 (54%)	Qualitative food insecurity N = 1775 (35%)	Quantitative food insecurity N = 560 (11%)
**Gender**				
Women	3017 (59.5%)	1691 (61.8%)	1056 (59.5%)	270 (48.2%)
Men	2051 (40.5%)	1042 (38.2%)	719 (40.5%)	290 (51.8%)
**Living with parents**				
Yes	3322 (65.5%)	2145 (78.5%)	917 (51.7%)	260 (46.4%)
No, but coming back on week-ends	292 (5.8%)	109 (4.0%)	153 (8.6%)	30 (5.4%)
No	1454 (28.7%)	479 (17.5%)	705 (39.7%)	270 (48.2%)
**Accommodation type**				
At their parents’ house	3322 (65.5%)	2145 (78.5%)	917 (51.7%)	260 (46.4%)
Living alone	527 (10.4%)	181 (6.6%)	261 (14.7%)	85 (15.2%)
Flat sharing	800 (15.8%)	324 (11.9%)	370 (20.8%)	106 (18.9%)
Collective residence	419 (8.3%)	83 (3.0%)	227 (12.8%)	109 (19.5%)
**Household cooking facilities**				
No food heating equipment	59 (1.2%)	5 (0.2%)	25 (1.4%)	29 (5.2%)
Food heating equipment only	385 (7.6%)	52 (1.9%)	202 (11.4%)	131 (23.4%)
Sufficient cooking equipment	4624 (91.2%)	2676 (97.9%)	1548 (87.2%)	400 (71.4%)
**Using food assistance**				
Never	4746 (93.6%)	2685 (98.2%)	1601 (90.2%)	460 (82.1%)
Less than 1/month	150 (3.0%)	30 (1.1%)	78 (4.4%)	42 (7.5%)
At least 1/ month	172 (3.4%)	19 (0.7%)	95 (5.4%)	58 (10.4%)
**Financial difficulties**				
1 – No difficulties	1534 (30.3%)	1340 (49.0%)	166 (9.4%)	28 (5.0%)
2	1242 (24.5%)	777 (28.4%)	414 (23.3%)	51 (9.1%)
3	1299 (25.6%)	451 (16.5%)	698 (39.3%)	150 (26.8%)
4	630 (12.4%)	128 (4.7%)	341 (19.2%)	161 (28.7%)
5 – Severe difficulties	363 (7.2%)	37 (1.4%)	156 (8.8%)	170 (30.4%)
**Having a job**				
No	3959 (78.1%)	2216 (81.1%)	1352 (76.2%)	391 (69.8%)
Less than 10 h/week	348 (6.9%)	202 (7.4%)	119 (6.7%)	27 (4.8%)
Between 10 h and 20 h/week	610 (12.0%)	255 (9.3%)	246 (13.9%)	109 (19.4%)
Over 20 h/week	151 (3%)	59 (2.2%)	58 (3.3%)	34 (6.0%)
**Enrolment at USPN**				
Re-enrolment	2395 (47.3%)	1272 (46.5%)	865 (48.7%)	258 (46.1%)
First enrolment	2673 (52.7%)	1461 (53.5%)	910 (51.3%)	302 (53.9%)
**Undergoing initial training**				
No (apprentice or professional resuming studies)	825 (16.3%)	499 (18.3%)	277 (15.6%)	49 (8.7%)
Yes	4243 (83.7%)	2234 (81.7%)	1498 (84.4%)	511 (91.3%)
**High-school diploma abroad**				
No	4072 (80.3%)	2429 (88.9%)	1299 (73.2%)	344 (61.4%)
Yes	996 (19.7%)	304 (11.1%)	476 (26.8%)	216 (38.6%)
**Academic discipline**				
Humanities, Languages and Social Sciences	1294 (25.5%)	636 (23.3%)	488 (27.5%)	170 (30.3%)
Health, Medicine and Human Biology	1693 (33.4%)	990 (36.2%)	559 (31.5%)	144 (25.7%)
Communication, Economic and Management sciences	541 (10.7%)	285 (10.4%)	193 (10.9%)	63 (11.1%)
Engineering sciences	602 (11.9%)	282 (10.3%)	240 (13.5%)	80 (14.3%)
University Institutes of Technology (technical studies)	938 (18.5%)	540 (19.8%)	294 (16.6%)	104 (18.6%)
**Study level**				
1^st^ year	1809 (35.7%)	994 (36.4%)	612 (34.5%)	203 (36.3%)
2^nd^ or 3^rd^ year	1965 (38.8%)	1070 (39.1%)	662 (37.3%)	233 (41.6%)
4^th^ year or over	1294 (25.5%)	669 (24.5%)	501 (28.2%)	124 (22.1%)
**Perceived academic dropout**				
No	4052 (80.0%)	2306 (84.4%)	1367 (77.0%)	379 (67.7%)
Yes	1016 (20.0%)	427 (15.6%)	407 (23.0%)	182 (32.3%)
**Company during meals**				
Eating alone	1718 (33.9%)	589 (21.6%)	780 (43.9%)	349 (62.3%)
Eating with someone	3350 (66.1%)	2144 (78.4%)	995 (56.1%)	211 (37.7%)
**Receiving food from family**				
Never	1304 (25.7%)	598 (21.9%)	470 (26.5%)	236 (42.1%)
Sometimes	1294 (25.5%)	421 (15.4%)	656 (37.0%)	217 (38.8%)
Often	2470 (48.7%)	1714 (62.7%)	649 (36.5%)	107 (19.1%)
**Cooking frequency**				
1 time/day or more	1875 (37.0%)	1079 (39.5%)	623 (35.1%)	173 (30.9%)
2 to 6 times/week	1435 (28.3%)	644 (23.5%)	616 (34.7%)	175 (31.3%)
1 time/week or less	849 (16.8%)	431 (15.8%)	270 (15.2%)	148 (26.4%)
Do not know	909 (17.9%)	579 (21.2%)	266 (15.0%)	64 (11.4%)

^a^ *Weighting calculated according to university official data on gender, initial training status, high-school diploma abroad, academic discipline, and study level.*

^b^ *p-values for chi-square test all < 0.01*

### Associations of FI with perceived academic dropout

**Fig 1:**
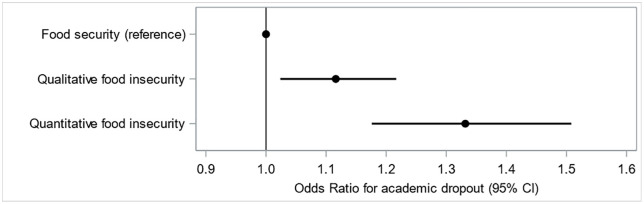
Associations of food insecurity with perceived academic dropout. CI: confidence interval. Model adjusted for gender, living with parents, household cooking facilities, enrolment at USPN, initial training status, high-school diploma abroad, academic discipline, study level, student job, financial difficulties, company for most meals, receiving food from family, using food assistance and cooking frequency.

In multivariate analyses, students experiencing qualitative (OR = 1.12 [1.02–1.22]) and quantitative (OR = 1.33 [1.18–1.51]) FI were more likely to report academic dropout (**Fig 1**).

### Associations of socioeconomic and demographic data with FI

In multivariable analyses (**Table 2**), men were more likely to report quantitative FI, but gender was not significantly associated with qualitative FI. Students living away from their parental home, living in a collective residence, lacking sufficient household cooking facilities, reporting financial difficulties, or relying on food assistance, were more likely to report both quantitative and qualitative FI. Students living alone or flat sharing were also more likely to report qualitative FI than those living with their parents. In gender-stratified analyses (**Table 3**), working less than 10 hours per week (compared to not having a job) was associated with lower quantitative FI in women and greater quantitative FI in men. Having a student job was not significantly associated with qualitative FI in either gender.

**Table 2:** Associations between university students’ characteristics and food insecurity (n = 5068).

	Quantitative food insecurity V*s food security* OR (95% CI)	Qualitative food insecurity V*s food security* OR (95% CI)	P-value ^*^
**Gender**			<0.0001
Women	1.00	1.00	
Men	1.41 (1.26-1.58)	1.06 (0.98-1.14)	
**Living with parents**			<0.0001
Yes	1.00	1.00	
No, but coming back on week-ends	1.45 (1.13-1.85)	2.52 (2.17-2.93)	
No	0.97 (0.83-1.13)	1.56 (1.41-1.73)	
**Accommodation type** ^**^			<0.0001
At their parents’ house	1.00	1.00	
Living alone	0.94 (0.77-1.14)	1.63 (1.43-1.86)	
Flat sharing	0.85 (0.72-1.02)	1.64 (1.47-1.83)	
Collective residence	1.92 (1.56-2.35)	2.56 (2.19-2.99)	
**Household cooking facilities**			<0.0001
No food heating equipment	6.75 (4.27-10.68)	2.21 (1.42-3.43)	
Food heating equipment only	3.99 (3.27-4.86)	2.43 (2.05-2.89)	
Sufficient cooking equipment	1.00	1.00	
**Using food assistance**			<0.0001
Never	1.00	1.00	
Less than 1/month	2.98 (2.25-3.95)	2.00 (1.59-2.53)	
At least 1/ month	3.74 (2.77-5.05)	2.83 (2.16-3.71)	
**Financial difficulties**			<0.0001
1 – No difficulties	1.00	1.00	
2	2.95 (2.34-3.72)	3.93 (3.55-4.34)	
3	12.70 (10.29-15.66)	10.43 (9.42-11.54)	
4	38.90 (31.14-48.59)	15.98 (14.03-18.20)	
5 – Severe difficulties	92.24 (70.83-120.12)	19.58 (16.07-23.85)	
**Enrolment at USPN**			0.001
Re-enrolment	1.00	1.00	
First enrolment	0.85 (0.75-0.96)	0.86 (0.79-0.93)	
**High-school diploma abroad**			<0.0001
No	1.00	1.00	
Yes	1.48 (1.28-1.72)	1.20 (1.09-1.34)	
**Study Level**			<0.0001
1^st^ year	2.26 (1.91-2.67)	1.49 (1.34-1.66)	
2^nd^ or 3^rd^ year	2.00 (1.69-2.37)	1.27 (1.14-1.42)	
4^th^ year or over	1.00	1.00	
**Company during meals**			<0.0001
Eating with someone	1.00	1.00	
Eating alone	2.97 (2.65-3.33)	1.76 (1.63-1.90)	
**Receiving food from family**			<0.0001
Never	1.00	1.00	
Sometimes	1.06 (0.93-1.22)	1.53 (1.38-1.69)	
Often	0.23 (0.20-0.27)	0.59 (0.54-0.65)	
**Cooking frequency**			<0.0001
1/day or more	1.00	1.00	
2 to 6/week	1.38 (1.21-1.58)	1.49 (1.37-1.62)	
1/week or less	2.28 (1.96-2.64)	1.35 (1.21-1.50)	
Does not know	0.91 (0.77-1.09)	1.09 (0.99-1.21)	

*CI: confidence interval. OR: odds ratio.*

* *The p-value represents the overall significance of each variable (type 3 analysis of effects) included in the multivariable multinomial logistic regression model, adjusted for gender, living with parents, household cooking facilities, enrolment at USPN, initial training status, high-school diploma abroad, academic discipline, study level, student job, financial difficulties, company for most meals, receiving food from family, using food assistance and cooking frequency.*

** *For the accommodation type, the fully adjusted model differs from the general fully adjusted model: the variable “living with parents” was changed for the variable “accommodation type” as adjustment variable.*

**Table 3:** Associations between university students’ characteristics and food insecurity, by gender (n = 5068).

	Women			Men		
	Quantitative Food Insecurity V*s Food Security* OR (95% CI)	Qualitative Food Insecurity *vs Food Security* OR (95% CI)	P-value *	Quantitative Food Insecurity *vs Food Security* OR (95% CI)	Qualitative Food Insecurity *vs Food Security* OR (95% CI)	P-value *
**Having a job**			0.05			0.001
No	1.00	1.00		1.00	1.00	
Less than 10h/week	0.57 (0.41-0.79)	0.92 (0.79-1.08)		1.88 (1.3-2.71)	1.16 (0.89-1.52)	
Between 10 h and 20 h/week	1.05 (0.85-1.3)	0.89 (0.77-1.02)		1.19 (0.94-1.5)	0.96 (0.8-1.14)	
Over 20 h/week	0.85 (0.57-1.25)	0.84 (0.63-1.11)		1.16 (0.79-1.7)	0.72 (0.52-1)	
**Undergoing initial training**			<0.0001			<0.0001
No (apprentice or professional resuming studies)	1.00	1.00		1.00	1.00	
Yes	6.85 (5.03-9.32)	1.46 (1.26-1.68)		0.54 (0.42-0.7)	0.63 (0.53-0.75)	

*CI: confidence interval. OR: odds ratio.*

*The p-value represents the overall significance of each variable (type 3 analysis of effects) included in the multivariable logistic regression model, adjusted for living with parents, household cooking facilities, enrolment at USPN, initial training status, high-school diploma abroad, academic discipline, study level, student job, financial difficulties, company for most meals, receiving food from family, using food assistance and cooking frequency.

### Associations of academic-related data with FI

Undergraduate students, those who had obtained their high-school diploma abroad, or who were re-enrolled at this university, were more likely to report quantitative and qualitative FI (**Table 2**). In gender-stratified analyses (**Table 3**), undergoing initial training was associated with greater qualitative and quantitative FI in women and lower FI in men.

### Associations of cooking and eating conditions with FI

Students usually eating alone or not cooking every day were more likely to report both quantitative and qualitative FI. Students often receiving food from family (compared to never) were less likely to report quantitative and qualitative FI. Those who sometimes received food from their family were more likely to report qualitative FI.

## Discussion

This cross-sectional study evaluated FI prevalence among university students from a socioeconomically disadvantaged area to assess the associations of FI with perceived academic dropout and to describe socioeconomic, demographic, and academic factors, together with cooking and eating conditions associated with FI in this population. As expected, given the local socioeconomic context, the student population included in our study reported high rates of both quantitative and qualitative FI (11% and 35%, respectively). These results align with previous results, including the systematic reviews of Nikolaus *et al*. (2020) [8], and Bruening *et al.* (2017) [13] of studies conducted primarily in the US, and reporting a mean prevalence of overall FI of 41% and 42%, respectively. In France in 2023, the National Observatory of Student Life, using the same assessment method as in our study, reported a prevalence of FI (either quantitative or qualitative) of 46% in a representative sample of 49,523 students [29]. Other studies conducted in two different universities in mainland France in 2021 found a lower FI prevalence of 43% (Grenoble, *n* = 4410) and 18% (Rouen, *n* = 3508), respectively [17,18]. Another study conducted in 2022 in a deprived French overseas region (French Guiana, **n* *= 286), reported a greater FI prevalence of 75% [19]. Across Europe, FI prevalence among students ranged from 28% in the UK (**n* *= 289) to 33% in Germany (**n* *= 547) and up to 82% in Greece (*n* = 236) [22–24]. These differences could be explained by the diversity of FI assessment methods. In both our study and in the National Observatory of Student Life [29], FI was assessed with a one-item question distinguishing between quantitative and qualitative FI. In other studies, FI was categorized by levels of severity, using questions from the FAO Food Insecurity Experience Scale Survey Module [18], the six-item Short Form of the US Food Security Module [17,19], or the US Household Food Insecurity Access Scale [22–24]. However, studies using similar assessment tools reported widely different prevalence rates of FI [17,19,22–24], suggesting that disparities in socioeconomic context may also explain these differences.

Importantly, the prevalence of quantitative FI reported in our study was three to four times higher than that assessed in the French adult population using the same method. Two studies conducted in 2008 and 2015 in a representative sample of French adults reported a prevalence of quantitative FI of 2.5% and 3.2%, respectively [27,33]. With underrepresentation of the most vulnerable groups in national studies [30], the higher prevalence of FI observed in university students is alarming insofar as this group represents about half of people aged 20–24 in France, and up to 75% of women and 65% of men aged 18–20 [34]. This large population is thus at risk of adverse physical and mental health outcomes [13,15]. Moreover, in line with previous work [12], our research shows that students experiencing FI are more likely to report academic dropout than their food-secure counterparts: 32% of students with quantitative FI reported dropping out, compared to 23% of those with qualitative FI, and 16% of food-secure students. Overall, our results highlight the significant burden of FI in the student population, exacerbating health and academic challenges for those already socioeconomically deprived.

Our study also describes the correlates of FI among university students. As previously reported in this population [12], the factors most strongly associated with FI were closely related to students’ socioeconomic status (i.e., financial difficulties and reliance on food assistance programmes). In our study, cooking facilities and accommodation type were also associated with FI, with students lacking sufficient cooking facilities, or living away from the family home, especially in collective residences, being more likely to experience both quantitative and qualitative FI. Previous results concerning accommodation type are not straightforward and may depend on the country of study. Living away from the family home is consistently associated with greater FI [35], and living in on-campus collective residences is usually associated with lower FI in the US [36]. This discrepancy with our findings seems likely due to differences between American and French higher education models, although such comparisons require caution. Living on-campus is often a preferred choice for students in the US and in the UK, whereas in France collective residences are a lower-cost accommodation option, with a smaller living space than other accommodation types [29].

Independent of these upstream factors, several interpersonal and individual factors were found to be associated with FI in our study. Students who reported receiving food from their family on a regular basis less often experienced either quantitative or qualitative FI, possibly due to increased food availability and budget reallocation. In France, where scholarships provide significant support for students [37], family support accounts for 41% of the average monthly income of students, including direct monetary aid and expenses paid by their family on their behalf [29]. In addition to family support, students who reported eating alone most of the time more often experienced quantitative and qualitative FI, echoing previous studies reporting associations between social isolation and FI among students [38]. International students more often reported FI in our study, irrespective of socioeconomic status and living conditions. Multiple interrelated factors may account for their difficulties in accessing sufficient and healthy food, including restricted access to rights and entitlements generally associated with permanent residence and citizenship, greater financial difficulties, barriers to obtaining culturally appropriate food, or an increased sense of isolation due to distance from family [21,29]. Not cooking every day was associated with greater quantitative and qualitative FI, suggesting that cooking could offer a solution to reduce FI. Finally, being a man was associated with greater quantitative FI, whereas gender was not significantly associated with qualitative FI.

Our results provide key insights to inform policies at universities across Europe that aim to reduce FI among students and so are of public health relevance. In particular, they help identify priority groups for targeted actions, and they underscore the importance of individual, interpersonal and environmental determinants of FI. Interestingly, universities’ databases record several factors that were found in our study to be associated with FI for all enrolled students, including gender, international status, training status, and study level. Universities could thus identify students most at risk of FI from their existing databases and consider implementing targeted prevention strategies. Asking a small set of additional questions about accommodation type, household cooking facilities, company during meals, frequency of cooking, and the Food Sufficiency Screener used to assess FI in our study would improve screening in the student population, guiding actions to reduce FI. These actions can be structural (social assistance, housing assistance, etc.), or more individual (e.g., cooking lessons), or of an interpersonal nature (e.g., reducing student loneliness). Importantly, university policies should go beyond emergency food assistance and consider preventive and multi-level approaches to FI. Because FI is also associated with academic dropout, actions could help to reduce inequalities in higher education and improve student retention.

### Strengths and limitations

The strengths of this study include its large sample size, the weighting procedure, and the consideration of several demographic, socioeconomic, and academic factors, and cooking and eating conditions. Disadvantaged higher education students are a hard-to-reach segment of the population [39]. This study provides valuable insights into the prevalence and correlates of FI in this population. However, some limitations should be mentioned. First, although a weighting procedure was conducted, this may have been insufficient to overcome selection bias fully. Extrapolation of the results to the general student population demands caution, because participants in nutrition research usually report more health-conscious behaviours and healthier dietary intakes overall than the general population [40]. Models accounted for many factors; however, residual factors (e.g., parents’ socio-economic status) cannot be entirely ruled out. Overall, our findings are likely most applicable to students in similarly disadvantaged contexts within Europe and may not be generalizable to other global settings. Second, FI is a sensitive issue, with possible bias from underreporting due to social desirability, or overreporting due to a student’s will to express their difficulties. Besides, the one-item screener used to assess FI does not capture the complexity of FI, which is a multidimensional effect [1]. More comprehensive tools such as the six-item Short Form of the US Food Security Module could enable a more accurate classification of FI severity [17,19]. This screener can, however, serve as a quick, low-burden tool, especially in large-scale surveys where time and resources are limited, and might be particularly useful for initial identification of at-risk individuals. Its previous use in nationally representative surveys also enables comparisons between student and general populations [27,33]. Finally, another important limitation of this study is its cross-sectional design, which limits our ability to establish causality between FI, academic dropout and socioeconomic variables. Following the same participants over time and assessing the longitudinal associations between initial FI and subsequent academic success, and between initial socioeconomic variables and subsequent FI status, would help to gain a better understanding of causal relationships.

In conclusion, this study confirms previous results that found high rates of FI among university students, highlighting this population as particularly at risk. Students reporting FI were also more likely to experience academic dropout, emphasizing that FI needs to be tackled for both public health and societal reasons. Several factors were associated with both quantitative and qualitative FI. These included being an undergraduate student or being re-enrolled at the same university, having obtained a high-school diploma abroad, living away from the parental home, living in a collective residence, reporting financial difficulties, relying on food assistance, never receiving food from family, usually eating alone, or not cooking daily. Individual, interpersonal, and environmental factors were therefore found to be associated with a greater risk of FI, suggesting that multi-level actions may be needed to address both upstream and individual determinants of FI. Future studies should further explore the role of the food environment in students’ FI by assessing associations between objective environmental characteristics (university restaurant opening hours, proximity to food outlets, food prices, institutional food policies, etc.) and students’ food security status.

## Supporting information

Table S1 Questionnaire. (DOCX)

Table S2 Characteristics of excluded and included participants. (DOCX)

Table S3 Unweighted data for characteristics of the sample of university students (n = 5068), by class of food security. (DOCX)
