# Supplementary material for: Correlates of food insecurity among university students in a socioeconomically disadvantaged area of the Paris suburbs: A cross-sectional study
Source: PLoS One. 2025 Oct 29;20(10):e0334523. doi: 10.1371/journal.pone.0334523 (PMC12571251; doi:10.1371/journal.pone.0334523)
Supplement: Table S1 — (DOCX) [file pone.0334523.s001.docx]

**Table S1:** Questionnaire

| **Question Title** | **Response Options** |
| --- | --- |
| Is this your first enrolment at USPN this year? | Yes; No |
| What is your status? | Student; Apprentice; Professional resuming studies |
| Did you come to France for your higher education after high school or a bachelor's degree (Campus France, Erasmus)? | Yes; No |
| You are | Female; Male; Prefer not to answer |
| So you are resuming studies. What is your training modality? | Professionalization contract; Continuing education; Validation of acquired experience |
| What is your UFR or Institute? | Training and research unit of Law, Political and Social Sciences; Training and research unit of Letters, Languages, Humanities and Societies; Training and research unit of Health, Medicine and Human Biology; UFR Economic and Management Sciences; Training and research unit of Communication Sciences; Galilee Institute; IUT of Villetaneuse; University Institute of Technology of Saint-Denis; University Institute of Technology of Bobigny; Success Support Service (university diploma) |
| What year are you in? | 1st year; 2nd or 3rd year; 4th year or more |
| Do you have a student job? | Yes; No; No but looking for one |
| How many hours does this job take per week? | less than 10h; 10 to 20h; more than 20h |
| Where do you currently live during a normal week of classes (Monday to Friday)? | With both parents; With my mother (or other female parent); With my father (or other male parent); In independent housing (rented, shared, owned, loaned); In collective housing (dormitory, boarding house, university residence, etc.); Elsewhere |
| If "Elsewhere", please specify | [Free text] |
| During a normal week of classes (Monday to Friday), you live: | Alone; In a couple; In a shared house; Other situation |
| If "Other situation", please specify | [Free text] |
| Do you sleep at your parents' or one of your parents' place on weekends? | Yes, every weekend; Yes, two to three weekends per month; Yes, one weekend per month; Yes, more rarely; No, never; Other |
| Your housing is: | A university residence or CROUS room; Another student residence; A boarding house; A dormitory; Other |
| If "Other", please specify | [Free text] |
| Can you have a meal in your housing? | Yes; No, there is neither a canteen nor cooking facilities |
| Under what conditions? | It is possible to cook; It is possible to reheat a meal but not to cook; There is a cafeteria, a canteen, a restaurant |
| Your housing is equipped with: |  |
| A refrigerator | Yes; No |
| A freezer (including refrigerator compartment) |  |
| A microwave oven |  |
| A conventional oven |  |
| Hotplates or a gas stove |  |
| A kettle |  |
| Storage spaces suitable for food |  |
| To what extent would you say you are facing financial difficulties at the moment on a scale of 1 to 5? | 1 (no financial difficulties); 2; 3; 4; 5 (very significant financial difficulties) |
| On average, what total budget do you allocate to your food per week (university restaurant, food shopping, dining out, etc.)? (in euros) | [Free text] |
| Since the beginning of the year, have you used food assistance? | Yes; No |
| How often do you use it? | Less than once a month; At least once a month |
| What type of association have you frequented? | Meal distribution; Distribution of baskets without choice; Social grocery stores or self-service; Distribution of vouchers, checks, reimbursements; Student association |
| Do family members help you by providing food supplies? | Never; Sometimes; Often |
| Among the following situations, which one best describes your current situation? | I often or sometimes don't have enough to eat; I have enough to eat, but not always the food I want; I can eat all the food I want |
| Have you ever had meals at the CROUS (restaurant, cafeteria)? | Yes; No |
| Are you satisfied with the services offered by the CROUS? | Yes; No |
| For what reasons? | [Free text] |
| In the last 15 days, how many times have you cooked a meal? | 3 times a day or more; 2 times a day; 1 time a day; 4 to 6 times a week; 2 to 3 times a week; 1 time a week; 1 time in the last 15 days; Never in the last 15 days; Don't know |
| In the last 15 days, how often have you had a meal in the following places? | (dropdown menu) |
| At home | 3 times a day or more; 2 times a day; 1 time a day; 4 to 6 times a week; 2 to 3 times a week; 1 time a week; 1 time in the last 15 days; Never in the last 15 days; Don't know |
| With family members (not living with you) |  |
| With friends |  |
| At the CROUS (restaurant, cafeteria) |  |
| In a collective restaurant other than the CROUS |  |
| In a fast-food restaurant (large chain, kebab, etc.) |  |
| At the restaurant |  |
| In the street, transport (including personal car) |  |
| Who do you most often have your meals with? | Alone; With people usually living with me (family, partner, roommates, children...); With friends, family (not living with you); With colleagues and/or other students; Don't know; Other |
| If "Other", please specify | [Free text] |
| Have you had distance learning courses? | Yes; No |
| Do you have difficulty following the courses? | Yes; No |
| Your difficulties in following the courses are due to (multiple choices possible): | Financial and/or material difficulties: housing, food, materials, etc.; Fragile health: illness, disability, psychological distress; Lack of method; Poor orientation; Other |
| Do you know which service to contact for help with these difficulties? | Yes; No |
| Do you think you are in a situation of "dropping out" of university? | Yes; No |

*CROUS: “*Centre Régional des Oeuvres Universitaires et Scolaires*“, i.e. Regional Centre for University and School Works.*

*For comparison purposes, questions were selected from three national surveys: the National Observatory of Student Life Survey (2023), which evaluates living and studying conditions of higher education students at the national level* [29]*; the “Food and nutritional status of food assistance beneficiaries” study (2011-2012), which was designed to describe the socio-demographic and economic profiles, food habits and nutritional status of food assistance beneficiaries* [30]*; and the Nutrition and Health Barometer (2008), a repeated cross-sectional survey carried out on representative samples of the French population* [27]*.*
