# Supplementary material for: Correlates of food insecurity among university students in a socioeconomically disadvantaged area of the Paris suburbs: A cross-sectional study
Source: PLoS One. 2025 Oct 29;20(10):e0334523. doi: 10.1371/journal.pone.0334523 (PMC12571251; doi:10.1371/journal.pone.0334523)
Supplement: Table S2 — (DOCX) [file pone.0334523.s002.docx]

**Table S2:** Characteristics of excluded and included participants

|  | **Excluded participants  n=1934 (28%)** | **Included participants  n= 5068 (72%)** | **P-value*** |
| --- | --- | --- | --- |
| **Food security class** |  |  | <0.001 |
| Food security | 511 (59%) | 2600 (51%) |  |
| Qualitative food insecurity | 254 (30%) | 1850 (37%) |  |
| Quantitative food insecurity | 95 (11%) | 618 (12%) |  |
| Missing data | 1074 | -- |  |
| **Gender** |  |  | <0.001 |
| Women | 1128 (58%) | 3352 (66%) |  |
| Men | 661 (34%) | 1716 (34%) |  |
| Do not wish to answer** | 145 (7.5%) | -- |  |
| Missing data | 0 | -- |  |
| **Living with parents** |  |  | <0.001 |
| Yes | 991 (76%) | 3134 (62%) |  |
| No, but coming back on week-ends | 56 (4.3%) | 299 (5.9%) |  |
| No | 256 (20%) | 1635 (32%) |  |
| Missing data | 631 | -- |  |
| **Accommodation type** |  |  | <0.001 |
| At their parents’ house | 991 (77%) | 3134 (62%) |  |
| Living alone | 87 (6.7%) | 564 (11%) |  |
| Flat sharing | 147 (11%) | 871 (17%) |  |
| Collective residence | 65 (5.0%) | 499 (9.8%) |  |
| Missing data | 644 | -- |  |
| **Household cooking facilities** |  |  | 0.5 |
| No food heating equipment | 12 (1.2%) | 57 (1.1%) |  |
| Food heating equipment only | 74 (7.4%) | 431 (8.5%) |  |
| Sufficient cooking equipment | 915 (91%) | 4580 (90%) |  |
| Missing data | 933 | -- |  |
| **Using food assistance** |  |  | <0.001 |
| Never | 972 (96%) | 4683 (92%) |  |
| Less than 1/month | 14 (1.4%) | 176 (3.5%) |  |
| At least 1/month | 29 (2.9%) | 209 (4.1%) |  |
| Missing data | 919 | -- |  |
| **Financial difficulties** |  |  | 0.03 |
| 1 – No difficulties | 326 (31%) | 1385 (27%) |  |
| 2 | 268 (25%) | 1230 (24%) |  |
| 3 | 251 (24%) | 1364 (27%) |  |
| 4 | 119 (11%) | 680 (13%) |  |
| 5 – Important difficulties | 88 (8.4%) | 409 (8.1%) |  |
| Missing data | 882 | -- |  |
| **Student job** |  |  | <0.001 |
| No | 1135 (81%) | 3774 (74.0%) |  |
| Less than 10h/week | 78 (5.6%) | 394 (7.8%) |  |
| Between 10 and 20h/week | 145 (10%) | 726 (14%) |  |
| Over 20h/week | 39 (2.8%) | 174 (3.4%) |  |
| Missing data | 537 | -- |  |
| **Enrolment at USPN** |  |  | <0.001 |
| First enrolment | 1101 (57%) | 2431 (48%) |  |
| Missing data | 0 | -- |  |
| **Undergoing initial training** |  |  | 0.02 |
| Yes | 1753 (91%) | 4685 (92%) |  |
| Missing data | 0 | -- |  |
| **High-school diploma abroad** |  |  | <0.001 |
| Yes | 368 (19%) | 1244 (25%) |  |
| Missing data | 36 | -- |  |
| **Academic discipline** |  |  | <0.001 |
| Humanities, Languages and Social Sciences | 443 (23%) | 1430 (28%) |  |
| Health, Medicine and Human Biology | 427 (22%) | 1270 (25%) |  |
| Communication, Economic and Management sciences | 211 (11%) | 668 (13%) |  |
| Engineering sciences | 214 (11%) | 802 (16%) |  |
| University Institutes of Technology | 639 (33%) | 898 (18%) |  |
| Missing data | 0 | -- |  |
| **Study Level** |  |  | <0.001 |
| 1^st^ year | 524 (27%) | 1340 (26%) |  |
| 2^nd^ or 3^rd^ year | 1042 (54%) | 2251 (44%) |  |
| 4^th^ year or over | 368 (19%) | 1477 (29%) |  |
| Missing data | 0 | -- |  |
| **Perceived academic dropout** |  |  | 0.40 |
| Yes | 136 (20%) | 1074 (21%) |  |
| Missing data | 1244 | -- |  |
| **Company for the majority of meals** |  |  | 0.002 |
| Eating alone | 185 (30%) | 1812 (36%) |  |
| Eating with someone | 442 (70%) | 3256 (64%) |  |
| Missing data | 1307 | -- |  |
| **Receiving food from family** |  |  | 0.01 |
| Never | 171 (21%) | 1310 (26%) |  |
| Sometimes | 237 (29%) | 1342 (26%) |  |
| Often | 413 (50%) | 2416 (48%) |  |
| Missing data | 1113 | -- |  |
| **Cooking frequency** |  |  | <0.001 |
| 1/day or more | 266 (31%) | 1883 (37%) |  |
| 2 to 6/week | 191 (22%) | 1484 (29%) |  |
| 1/week or less | 122 (14%) | 843 (17%) |  |
| Does not know | 290 (33%) | 858 (17%) |  |
| Missing data | 1065 | -- |  |

***** *P-value represented the comparison between included and excluded participants across variables (Chi-square test).*

** *Students who chose not to specify their gender were excluded, since margin calibration was made on gender’s data from the university, which included only women and men.*
