# Supplementary material for: Correlates of food insecurity among university students in a socioeconomically disadvantaged area of the Paris suburbs: A cross-sectional study
Source: PLoS One. 2025 Oct 29;20(10):e0334523. doi: 10.1371/journal.pone.0334523 (PMC12571251; doi:10.1371/journal.pone.0334523)
Supplement: Table S3 — (DOCX) [file pone.0334523.s003.docx]

**Table S3:** Unweighted data for characteristics of the sample of university students (n=5068), by class of food security

|  | **Raw data** | | |
| --- | --- | --- | --- |
|  | **Food security**  n=2600 (51%) | **Qualitative food insecurity**  n=1850 (37%) | **Quantitative food insecurity**  n=618 (12%) |
| **Gender** |  |  |  |
| Women | 1771 (68.1%) | 1226 (66.3%) | 355 (57.4%) |
| Men | 829 (31.9%) | 624 (33.7%) | 263 (42.6%) |
| **Living with parents** |  |  |  |
| Yes | 2003 (77%) | 887 (48%) | 244 (39.5%) |
| No, but coming back on week-ends | 104 (4%) | 161 (8.7%) | 34 (5.5%) |
| No | 493 (19%) | 802 (43.4%) | 340 (55%) |
| **Accommodation type** |  |  |  |
| At their parents’ house | 2003 (77%) | 887 (48%) | 244 (39.5%) |
| Living alone | 177 (6.8%) | 285 (15.4%) | 102 (16.5%) |
| Flat sharing | 317 (12.2%) | 414 (22.4%) | 140 (22.7%) |
| Collective residence | 103 (4%) | 264 (14.3%) | 132 (21.4%) |
| **Household cooking facilities** |  |  |  |
| No food heating equipment | 7 (0.3%) | 20 (1.1%) | 30 (4.9%) |
| Food heating equipment only | 58 (2.2%) | 222 (12%) | 151 (24.4%) |
| Sufficient cooking equipment | 2535 (97.5%) | 1608 (86.9%) | 437 (70.7%) |
| **Using food assistance** |  |  |  |
| Never | 2538 (97.6%) | 1653 (89.4%) | 492 (79.6%) |
| Less than 1/month | 37 (1.4%) | 85 (4.6%) | 54 (8.7%) |
| At least 1/month | 25 (1%) | 112 (6.1%) | 72 (11.7%) |
| **Financial difficulties** |  |  |  |
| 1 – No difficulties | 1200 (46.2%) | 160 (8.7%) | 25 (4.1%) |
| 2 | 762 (29.3%) | 418 (22.6%) | 50 (8.1%) |
| 3 | 470 (18.1%) | 728 (39.4%) | 166 (26.9%) |
| 4 | 129 (5%) | 376 (20.3%) | 175 (28.3%) |
| 5 – Important difficulties | 39 (1.5%) | 168 (9.1%) | 202 (32.7%) |
| **Student job** |  |  |  |
| No | 2013 (77.4%) | 1346 (72.8%) | 415 (67.2%) |
| Less than 10h/week | 231 (8.9%) | 134 (7.2%) | 29 (4.7%) |
| Between 10 and 20h/week | 288 (11.1%) | 302 (16.3%) | 136 (22%) |
| Over 20h/week | 68 (2.6%) | 68 (3.7%) | 38 (6.2%) |
| **Enrolment at USPN** |  |  |  |
| Re-enrolment | 1366 (52.5%) | 965 (52.2%) | 306 (49.5%) |
| First enrolment | 1234 (47.5%) | 885 (47.8%) | 312 (50.5%) |
| **Undergoing initial training** |  |  |  |
| No (apprentice or professional resuming studies) | 230 (8.9%) | 128 (6.9%) | 25 (4.1%) |
| Yes | 2370 (91.2%) | 1722 (93.1%) | 593 (96%) |
| **High-school diploma abroad** |  |  |  |
| No | 2238 (86.1%) | 1244 (67.2%) | 342 (55.3%) |
| Yes | 362 (13.9%) | 606 (32.8%) | 276 (44.7%) |
| **Academic discipline** |  |  |  |
| Humanities, Languages and Social Sciences | 690 (26.5%) | 554 (30%) | 186 (30.1%) |
| Health, Medicine and Human Biology | 738 (28.4%) | 419 (22.7%) | 113 (18.3%) |
| Communication, Economic and Management sciences | 338 (13%) | 243 (13.1%) | 87 (14.1%) |
| Engineering sciences | 350 (13.5%) | 338 (18.3%) | 114 (18.5%) |
| University Institutes of Technology | 484 (18.6%) | 296 (16%) | 118 (19.1%) |
| **Study level** |  |  |  |
| 1^st^ year | 737 (28.4%) | 458 (24.8%) | 145 (23.5%) |
| 2^nd^ or 3^rd^ year | 1170 (45%) | 777 (42%) | 304 (49.2%) |
| 4^th^ year or over | 693 (26.7%) | 615 (33.2%) | 169 (27.4%) |
| **Perceived academic dropout** |  |  |  |
| No | 2165 (83.3%) | 1408 (76.1%) | 421 (68.1%) |
| Yes | 435 (16.7%) | 442 (23.9%) | 197 (31.9%) |
| **Company for the majority of meals** |  |  |  |
| Eating alone | 595 (22.9%) | 820 (44.3%) | 397 (64.2%) |
| Eating with someone | 2005 (77.1%) | 1030 (55.7%) | 221 (35.8%) |
| **Receiving food from family** |  |  |  |
| Never | 549 (21.1%) | 494 (26.7%) | 267 (43.2%) |
| Sometimes | 410 (15.8%) | 688 (37.2%) | 244 (39.5%) |
| Often | 1641 (63.1%) | 668 (36.1%) | 107 (17.3%) |
| **Cooking frequency** |  |  |  |
| 1 time/day or more | 1041 (40%) | 653 (35.3%) | 189 (30.6%) |
| 2 to 6 times/week | 613 (23.6%) | 660 (35.7%) | 211 (34.1%) |
| 1 time/week or less | 399 (15.4%) | 286 (15.5%) | 158 (25.6%) |
| Do not know | 547 (21%) | 251 (13.6%) | 60 (9.7%) |
